# Review of the Most Cited Patient-Reported Outcome Measure (PROM) Studies Published in the Neurospine Surgical Literature

**DOI:** 10.7759/cureus.44262

**Published:** 2023-08-28

**Authors:** Abdulhakim B Jamjoom, Abdulhadi Y Gahtani, Moajeb T Alzahrani, Ahmad S Albeshri, Momen A Sharab

**Affiliations:** 1 Neurosurgery, King Saud Bin Abdulaziz University for Health Sciences College of Medicine, Jeddah, SAU; 2 Neurosurgery, King Abdulaziz Medical City Western Region, Jeddah, SAU

**Keywords:** citation rates, survey questionnaires, bibliometrics, spine journals, neurosurgical journals, proms, patient-reported outcome measures

## Abstract

Patient-reported outcome measures (PROMs) are validated tools that are widely utilized in research and patient care. Their diversity, quality, and application remain matters of peak research interest. This article is a review of the PROMs that were utilized in high-impact publications in the neurospine surgical literature. The 50 most cited articles on the subject were selected and analysed. Most (42 articles) were published in spine journals and, in particular, in the journal Spine (Phila Pa 1976) (28 articles). A total of 34 PROMs were utilized, of which 24 were used only once in single studies. The four most common PROMs were Scoliosis Research Society-22 (SRS-22) (15 articles), Short Form-12 and Short Form-36 (SF-12 and SF-36) (11 articles), Ronald-Morris Disability Questionnaire (RMDQ) (nine articles), and Oswestry Disability Index (ODI) (five articles). Nineteen articles focused on validating translated versions of 11 PROMs to other languages. The languages that had the maximal number of tools translated to amongst the highly cited articles were Italian (six tools), Portuguese (four tools), German (three tools), and Japanese (three tools). The most common diagnoses and the PROMs used for them were back pain and cervical spine disorder (SF-12 and SF-36 (nine articles), RMDQ (eight articles), and ODI (five articles)), and idiopathic scoliosis (SRS-22) (14 articles)). The median (range) article citation number was 137 (78-675). The four most cited PROMs were SRS-22 (2,869), SF-12 and SF-36 (2,558), RMDQ (1,456), and ODI (852). Citation numbers were positively impacted by article age and participant number but not by tool type or clinical diagnosis. In conclusion, a wide range of PROMs was utilized in the 50 most cited publications in the neurospine surgical literature. The majority were disease-specific rather than generic and targeted particular spine pathology. Neurosurgical PROMs were under-represented amongst the most cited articles. Awareness of the PROMs used in high-impact studies may be helpful in tool selection in future research. PROMs are valuable in standardizing subjective outcomes. Their use in research and clinical settings in any validated language is highly encouraged.

## Introduction and background

Patient-reported outcome measures (PROMs) are validated questionnaires, also referred to as instruments or tools, that are completed by patients and used to assess their perceptions of their quality of life (QOL) [1]. PROMs are generally recognized as key tools in research, clinical decision-making, patient-centered care, health policy, and more recently reimbursement rulings [1-3]. They are designed to be either disease-specific or generic. Disease-specific PROMs are used in evaluating the impact of a certain disease or condition. Generic PROMs, on the other hand, provide a more general assessment of the health of the individual within certain dimensions, which include physical function, social function, pain, and depression or anxiety [1,2]. For PROMs to be useful in research and patient care, they must possess certain quality properties, such as reliability, validity, and responsiveness [1-3]. There is also an increasing recognition that new tools should be based on clinimetric rather than psychometric criteria [4]. Evidence in the literature suggests that the number of PROMs grew substantially between the 1980s and 2000s, but slowed more recently. The number of publications discussing PROMs, however, continued to increase [1].

Numerous PROMs were utilized in studies that were published in spine journals. Guzman et al. [5] found 206 PROMs that were used in articles that were published in five spine journals during 2004-2013. In addition, Ramasamy et al. [6] stated that 176 PROMs were used to assess chronic back pain in the literature during 2011 and 2015. Furthermore, Beighley et al. [7] identified 37 spine-specific PROMs that were used in 8,599 articles. The application of PROMs in neurosurgical research has been less common. Ghimire et al. [8] detected only 26 PROMs in 137 articles that were published in the neurosurgical literature from 1806 to 2016. Also, Hansen et al [9] reported 46 PROMs in 31 studies that were published in three pediatric neurosurgery journals from 2005 to 2014. The diversity in the numbers and types of PROMs used in spine and neurosurgical publications, and the increasing popularity of bibliometric reviews of high-impact studies [10], motivated us to do this review. It was felt that drawing attention to the PROMs that were utilized in high-impact spine and neurosurgical studies may be useful in enhancing their selection and utilization in future research. This review aimed at identifying and reviewing the PROMs that were used in the 50 most cited publications in the neurospine surgical literature.

## Review

Methods

This study was carried out at King Saud Bin Abdulaziz University for Health Science (KSAU-HS), Jeddah, Saudi Arabia. No ethical approval was necessary by our institution as the study was based on data obtained from open-access sources. The PubMed database was searched in March 2023 for studies that used PROMs and were published in the neurospine surgical literature. The search was carried out using the keywords alone or in combination that are shown in Table 1.

**Table 1:** The keywords used in the PubMed search methods to identify the 50 most cited patient-reported outcome studies in the neurospine surgical literature

The PubMed Search
[Title] Patient-Reported Outcome Measures, PROMs, Patients, Survey, Questionnaire
AND
[Journal] The following thirty individual neurosurgical and spine journals by name:
Spine (Phila Pa 1976)
The Spine Journal
European Spine Journal
Joint Bone Spine
Spinal Cord
Journal of Neurology Neurosurgery and Psychiatry
Journal of Neurosurgery
Journal of Neurosurgery Spine
Journal of Neurosurgery Pediatrics
Neurosurgery
World Neurosurgery
Acta Neurochirurgica
British Journal of Neurosurgery
Neurosurgical Review
Neurosurgical Focus
Surgical Neurology
Surgical Neurology International
Clinical Neurology and Neurosurgery
Clinical Neurosurgery
Child's Nervous System
Pediatric Neurosurgery
Stereotactic and Functional Neurosurgery
Journal of Neurosurgical Sciences
Journal of Neurological Surgery Part B Skull Base
Journal of Neurological Surgery Part A Central European Neurosurgery
Neurospine
Asian Journal of Neurosurgery
Journal of Korean Neurosurgical Society
Neurologia Medico-Chirurgica
Pituitary

The search yielded a total of 1,217 articles. The inclusion criteria in this review were highly cited publications in the neurospine surgical literature that utilized PROMs. Studies in which no PROMs were used, and the participants were not patients or did not provide adequate data were excluded. Using Google Scholar, citation numbers for the 1,217 articles were documented. In view of the regular changes in the citation numbers, the search findings on a single day (June 1, 2023) were documented and used for analysis. The 50 articles with the highest citation numbers were identified and selected. Using full articles, the relevant data were collected by two of the authors independently, and any discrepancies were resolved by consensus. The following parameters were collected for each article: year of publication, publishing journal, its impact factor (IF), number of authors, number of centres, first author’s country, PROMs type, number, and whether the tool was a translation to another language, number of participating patients, their response rate, the diagnoses for which the PROMs were used, and whether the recruitment was from a single country or multiple countries. The journal impact factor (IF) data were obtained from an online source [11]. The statistical analysis was carried out by correlating the total citation number for the selected articles with various publications, PROMs, and participants-related parameters. The correlation analysis was done by calculating the Pearson correlation coefficient (R) using social sciences statistics [12], and significance was determined when p≤0.05.

Results

The 50 most cited publications in the neurospine surgical literature that utilized PROMs are analysed in Table 2 [13-62].

**Table 2:** The 50 most cited patient-reported outcome measure (PROM) studies published in the neurospine surgical literature Abbreviations: [SRS-22] Scoliosis Research Society-22, [SF-12 & 36] Short Form-12 and 36, [PDQLQ] Parkinson Disease Quality of Life Questionnaire, [CREST] Clinical Rehabilitation Using Electrical Stimulation Via Telematics, [QOL] Quality of Life, [Q] Questionnaire, [ODI] Oswestry Disability Index, [RMDQ] Ronald-Morris Disability Questionnaire, [JOA] Japanese Orthopaedic Association, [P&DQ] Pain and Dysthesia Questionnaire, [SF36-MHS] Short Form-36 Mental Health Scale, [SF36-BPS] Short Form-36 Bodily Pain Scale, [CIQ] Community Integration Questionnaire, [BIPS] Brief Pain Inference Scale, [MSQOL-54] Multiple Sclerosis Quality of Life-54 (SF-36 & MS-18), [FABQ] Fear Avoidance Belief Questionnaire, [V&M NDI] Vernon and Mior Neck Disability Index, [TSKP] Tampa Scale of Kinesiophobia, [RHFUQ] Rivermead Head Injury Follow up Questionnaire, [FRI] Functional Rating Index, [NSQ] Nordic Sleep Questionnaire, [PSAQ] Patient Specific Activity Questionnaire, [MSS] McGill Sensory Score, [MES] McGill Effective Score, [PES] Prolo Economic Score, [PFS] Prolo Functional Score, [MRPDS] Modified Ransford Pain Drawing Score, [HOQ] Hydrocephalus Outcome Questionnaire, [VAS] Visual Analogue Scale, [MDI] Myelopathy Disability Index, [HADS] Hospital Anxiety and Depression Scale, [DSQ], Dysphagia Short Questionnaire, [HAQ] Health Assessment Questionnaire, [F-DASH-D/S] French Disability of the Arm, Shoulder, and Hand Questionnaire- Disability/Symptom, [SAQ] Spinal Appearance Questionnaire, and [SCI] Spinal Cord Injury

No.	First Author [Ref]	Year	No. Patients	PROMs Tools	Study Focus	Cites
1	Asher M [13]	2003	83	SRS-22, SF-36	Validation of SRS-22 in scoliosis	675
2	de Boer AG [14]	1996	529	PDQOLQ	Validation of QOLQ in Parkinson's disease	492
3	Snoek GJ [15]	2004	5,500	CREST	Impact of Hand function on QOL in spinal cord injury	490
4	Fujiwara A [16]	2003	97	ODI, RMDQ, SF36, JOA	Validation of the Japanese version of ODI & RMDQ in back pain	376
5	Luo X [17]	2003	2,520	SF-12	Validation of SF-12 in back pain	348
6	Finnerup NB [18]	2001	436	P&DQ	Neuropathic pain in spinal cord injury	332
7	Jensen MP [19]	2005	339	SF36-MHS, CIQ, SF36-BPS, BIPS	Chronic pain in spinal cord injury	314
8	Asher M [20]	2003	119	SRS-22	Validation of discriminating capacity of SRS-22 in scoliosis	307
9	Asher M [21]	2003	61	SRS-22	Validation of responsiveness of SRS-22 to changes in scoliosis	291
10	Asher M [22]	2006	111	SRS-22	Validation of refinement of SRS-22 for patients below 18 in scoliosis	262
11	Wiesinger GF [23]	1999	125	RMDQ	Validation of the German version of RMDQ in back pain	247
12	Alanay A [24]	2005	82	SRS-22	Validation of the Turkish version of SRS-22 in scoliosis	224
13	Solari A [25]	1999	219	MSQOL-54	Validation of the Italian version of MSQOL-54 in Multiple Sclerosis	223
14	Chaory K [26]	2004	248	FABQ	Validation of the French version of FABQ in back pain	219
15	McCarthy MJ [27]	2007	160	V&M NDI, SF-36	Validation of V & M NDI in neck pain	213
16	Ravenscroft A [28]	2000	216	Authors' Pain Q	Chronic pain in spinal cord injury	192
17	Staerkle R [29]	2004	388	FABQ	Validation of the Swiss German version of FABQ in back pain	190
18	de Souza FS [30]	2008	50	FABQ, TSKP	Validation of Brazil Portuguese versions of FABQ & TSKP in back pain	180
19	Monticone M [31]	2012	215	ODI, RMDQ	Validation of the Italian version of ODI & RMDQ in back pain	175
20	Crawford S [32]	1996	97	RHFUQ	Validation of RHFUQ in traumatic brain injury	173
21	Costa LO [33]	2007	140	RMDQ, FRI	Validation of the Brazil Portuguese version of RMDQ & FRI in back pain	169
22	Hagell P [34]	2007	257	PDQOL-39	Validation of PDQOL-39 in Parkinson's disease	162
23	Cheung KM [35]	2007	99	SRS-22	Validation of the Chinese version of SRS-22 in scoliosis	156
24	Bago J [36]	2004	175	SRS-22	Validation of the Spanish version of SRS-22 in scoliosis	143
25	Biering-Sørensen F [37]	2001	487	NSQ	Sleep disturbance in spinal cord injury	139
26	Gil Z [38]	2004	64	Skull base cancer QOLQ	Validation of QOLQ in anterior skull base cancer	135
27	BenDebba M [39]	2002	216	Cervical Spine Outcomes Q	Validation of Cervical Spine Outcomes Questionnaire in neck pain	134
28	Climent JM [40]	2005	175	SRS-22	Validation of the Spanish version of SRS-22 in scoliosis	131
29	Asher M [41]	2004	67	SRS-22	Impact of deformity on preop QOL in scoliosis	130
30	Singh A [42]	2006	105	SF-12, SF-36	Validation of SF-12 & SF-36 in cervical myelopathy	130
31	Frost H [43]	2008	201	PSAQ, ODI, RMDQ	Validation of PSAQ responsiveness to patients' changes in back pain	125
32	Monticone M [44]	2010	223	TSKP	Validation of the Italian version of TSKP in back pain	124
33	Gatchel RJ [45]	1999	146	SF-36	SF-36 responsiveness after functional restoration in SCI	122
34	Hashimoto H [46]	2007	172	SRS-22	Validation of the Japanese version of SRS-22 in scoliosis	122
35	Voorhies RM [47]	2007	121	VAS, MSS, MES, PES, PFS, MRPDS	Outcome of surgical treatment for back pain	108
36	Kulkarni AV [48]	2004	69	Hydrocephalus Outcome Q	Validation of HOQ	107
37	Guilfoyle MR [49]	2009	620	SF36, VAS, RMDQ, MDI, HADS	Validation of SF36 in neck and back pain	101
38	Bagó J [50]	2009	97	SRS-22	Minimal important differences in SRS-22 after surgery in scoliosis	99
39	Padua R [51]	2002	70	RMDQ, SF-36, VAS	Validation of Italian versions of RMDQ in back pain	98
40	Helenius I [52]	2006	84	ODI, SRS-22	Outcome of surgery in spondylolisthesis	97
41	Walsh TL [53]	2006	420	SF36	Validation of Mental Component Summary in SF-36 in back pain	94
42	Turner JA [54]	2003	309	RMDQ, SF36, SF12	Validation of RMDQ in back injury claimants	85
43	Skeppholm M [55]	2012	101	Dysphagia Short Q	Validation of DSQ in anterior cervical spine surgery	85
44	El Meidany YM [56]	2003	184	Health Assessment Q	Validation of Arabic version of HAQ in Rheumatoid Arthritis	85
45	Fayad F [57]	2008	150	F-DASH-D/S	Validation of French version of DASH-D/S in shoulder disorders	83
46	Parent EC [58]	2009	383	SRS-22	Validation of discriminating capacity of SRS-22 in scoliosis	82
47	Niemeyer T [59]	2009	222	SRS-22, RMDQ	Validation of German version of SRS-22 in scoliosis	80
48	Carreon LY [60]	2011	1,802	Spinal Appearance Q	Validation of SAQ of SRS-22 in scoliosis	79
49	Payares K [61]	2011	111	ODI	Validation of Colombian version of ODI in back pain	79
50	Vandervelde L [62]	2010	248	ABILHAND	Validation of ABILHAND in children and adults with neuromuscular disease	78

The median (range) number of PROMs per article was 1 (1- 6) with 14 studies using more than one tool. The median (range) article citation number was 137 (78-675). The number of citations for the 50 articles was 9,285. The median (range) article publication year and age were 2005 (1996-2012) and 18 (11-27) years, respectively. The median (range) publishing journal’s IF was 3.24 (1.12-13.65). The median (range) number of authors and centres per article were 5 (2-12) and 2 (1-6), respectively. The publishing journals and first authors’ countries are shown in Table 3.

**Table 3:** Distribution of the 50 most cited PRO articles and their citations according to the publishing journals and the first author’s country Others*: Austria, Switzerland, Brazil, Australia, China, Israel, Finland, Egypt, Germany, Columbia, Belgium

Features		Total Articles	Total Cites
Publishing Journal	Spine (Phila Pa 1976)	28	5,202
	Spinal Cord	5	1,467
	Journal of Neurology Neurosurgery and Psychiatry	5	1,128
	European Spine Journal	5	775
	Journal of Neurosurgery	2	242
	The Spine Journal	2	202
	Joint Bone Spine	2	168
	British Journal of Neurosurgery	1	101
First Author’s Country	USA	13	2,949
	UK	6	934
	Italy	4	620
	Spain	3	373
	Netherlands	2	982
	France	2	302
	Denmark	2	471
	Japan	2	498
	Canada	2	189
	Sweden	2	247
	Others*	12	1,720

Most articles were published in spine journals (42 articles), in particular in the journal Spine (Phila Pa 1976) (28 articles). The three countries with the highest number of first authorship articles and citation numbers were the USA (13 articles, 2,949 citations), the UK (six articles, 934 citations), and Italy (four articles, 620 citations). A total of 34 different tools were used, of which 26 were employed once in single studies. The distribution of articles according to the PROMs, their citations, and translation to other languages is illustrated in Table 4.

**Table 4:** Distribution of the 50 most cited PROM articles according to the utilized tools, citations, and translated versions. Some articles used more than one PROM tool Miscellaneous:* Clinical Rehabilitation Using Electrical Stimulation Via Telematics, Japanese Orthopaedic Association, Pain and Dysthesia Questionnaire, Community Integration Questionnaire, Brief Pain Inference Scale, Vernon and Mior Neck Disability Index, Authors’ Pain Questionnaire, Rivermead Head Injury Follow up Questionnaire, Nordic Sleep Questionnaire, Skull Base Cancer Quality of Life Questionnaire, Cervical Spine Outcome Questionnaire, Patient-Specific Activity Questionnaire, McGill Sensory Score, McGill Affective Score, Prolo Economic Score, Prolo Functional Score, Modified Ransford Pain Drawing Score, Hydrocephalus Outcome Questionnaire, Myelopathy Disability Index, Hospital Anxiety and Depression Scale, Dysphagia Short Questionnaire, and ABILHAND

PROMs Tool Types [Ref]	Total Articles	Total Cites	Languages of PROMS Translated Versions [Ref]
Scoliosis Research Society-22 [13,20,21,22,24,25,40,41,46,50,52,58,59,60]	15	2,869	German [59], Japanese [46], Spanish [36,40], Chinese [35], Turkish [24]
Short Form 12 and Short Form-36 [13,16,17,19,27,42,45,49,51,53,54]	11	2,558	Italian [51]
Ronald-Morris Disability Questionnaire [16,23,31,33,43,49,51,54,59]	9	1,456	German [23], Italian [31,51], Japanese [16], Portuguese [33]
Oswestry Disability Index [16,31,43,52,61]	5	852	Italian [31], Japanese [16], Colombian [61]
Fear Avoidance Belief Questionnaire [26,29,30]	3	589	German [29], French [26], Portuguese [30]
Visual Analogue Scale [47,49,51]	3	307	Italian [51]
Parkinson Disease Quality of Life Questionnaire [14,34]	2	654	None
Tampa Scale of Kinesiophobia [30,44]	2	304	Italian [44], Portuguese [30]
Multiple Sclerosis Quality of Life-54 [25]	1	223	Italian [25]
Functional Rating Index [33]	1	169	Portuguese [33]
Health Assessment Questionnaire [56]	1	85	Arabic [56]
Disability of the Arm, Shoulder, and Hand Questionnaire Disability/Symptom [57]	1	83	French [57]
Miscellaneous* [15,16,18,19,27,28,32,37,38,39,43,47,48,49,55,62]	16	3,103	None

The PROMs that were utilized most were Scoliosis Research Society-22 (SRS-22) (15 articles), Short Form 12 and Short Form-36 (SF-12 and SF-36) (11 articles), RMDQ (nine articles), ODI (five articles), Fear Avoidance Belief Questionnaire (FABQ) (three articles), and Visual Analogue Scale (VAS) (three articles). The four PROMs utilized in the articles that received the highest numbers of total citations were SRS-22 (2,869), SF-12 and SF-36 (2,558), RMDQ (1,456), and ODI (852). Validation of the translation of 11 PROMs was the focus of the study in 19 articles. The languages that had the maximal number of tools translated to amongst the highly cited articles were as follows: Italian: six tools (SF-36, RMDQ, ODI, VAS, Tampa Scale of Kinesiophobia (TSKP), Multiple Sclerosis Quality of Life-54 (MSQOL-54)); Portuguese: four tools (RMDQ, FABQ, TSKP, Functional Rating Index (FRI)); German: three tools (SRS-22, RMDQ, FABQ); Japanese: three tools (SRS-22, RMDQ, ODI); and French: two tools (FABQ, Disability of the Arm, Shoulder, and Hand Questionnaire- Disability/Symptom (DASH-D/S)). All the utilized PROMs were disease-specific, except two (SF-12 or 36 and Health Assessment Questionnaire (HAQ)), which were considered generic. The median (range) number of participating patients was 174 (50-5,500). The mean and median (range) response rates were 82% and 88% (21%-100%), respectively. The participating patients were from one country, except in seven studies where they were from two to seven countries. The distribution of the PROMs according to 12 clinical diagnoses is summarized in Table 5.

**Table 5:** Distribution of the 50 most cited PROM articles according to the clinical diagnoses and the utilized PROMs. Some articles used more than one PROM tool Others1: JOA, FRI, PSAQ, MSS, MES, PES, PFS, MRPDS, MDI, HADS, V&M NDI, CSOQ, DSQ. Others2: CREAST, CIQ, BIP, NSQ, P&DQ, APQ. Abbreviations: [SRS-22] Scoliosis Research Society-22, [SF-12 and SF-36] Short Form 12 and Short Form-36, [RMDQ] Ronald-Morris Disability Questionnaire, [ODI] Oswestry Disability Index, [FABQ] Fear Avoidance Belief Questionnaire, [VAS] Visual Analogue Scale, [TSKP] Tampa Scale of Kinesiophobia, [PDQOLQ] Parkinson Disease Quality of Life Questionnaire, [MSQOL-54] Multiple Sclerosis Quality of Life-54, [RHIFUQ] Rivermead Head Injury Follow up Questionnaire, [SBQOLQ] Skull Base Quality of Life Questionnaire, [HOQ] Hydrocephalus Outcome Questionnaire, [HAQ] Health Assessment Questionnaire, [DASH-D/S] Disability of the Arm, Shoulder, and Hand Questionnaire- Disability/Symptom, [JOA] Japanese Orthopaedic Association, [FRI] Functional Rating Index, [PSAQ] Patient Specific Activity Questionnaire, [MSS] McGill Sensory Score, [MES] McGill Effective Score, [PES] Prolo Economic Score, [PFS] Prolo Functional Score, [MRPDS] Modified Ransford Pain Drawing Score, [MDI] Myelopathy Disability Index, [HADS] Hospital Anxiety and Depression Scale, [V&M NDI] Vernon and Mior Neck Disability Index, [CSOQ] Cervical Spine Outcomes Questionnaire, [DSQ] Dysphagia Short Questionnaire, [CREST] Clinical Rehabilitation Using Electrical Stimulation Via Telematics, [CIQ] Community Integration Questionnaire, [BIPS] Brief Pain Inference Scale, and [NSQ] Nordic Sleep Questionnaire, [P&DQ] Pain & Dysthesia Questionnaire, and [APQ] Author’s Pain Questionnaire

Clinical Diagnoses [Ref]	Total Articles	Total Cites	Utilized PROMs Tools		
			Tools Type	Tools Articles	Tool Cites
Low Back Pain and Cervical Spine Disorder [16,17,23,26,27,29,30,31,33,39,42,43,44, 47,49,51,52,53,54,55,61]	21	3499	SF-12 and SF-36	9	1,567
			RMDQ	8	1,376
			ODI	5	852
			FABQ	3	589
			VAS	3	307
			TSKP	2	304
			SRS-22	1	97
			Others^1^	8	1,311
Idiopathic Scoliosis [13,20,21,22,24,35,36,40,41,46,50,58,59,60]	14	2781	SRS-22	14	2,781
			SF-36	1	675
			RMDQ	1	80
Spinal Cord Injury [15,18,19,28,37,45]	6	1467	SF-36	1	314
			Others^2^	5	1,467
Parkinson Disease [14,34]	2	654	PDQOLQ	2	654
Multiple Sclerosis [25]	1	223	MSQOL-54	1	223
Traumatic Brain Injury [32]	1	173	RHIFUQ	1	173
Skull Base Cancer [38]	1	135	SBQOLQ	1	135
Hydrocephalus [48]	1	107	HOQ	1	107
Rheumatoid Arthritis [56]	1	85	HAQ	1	85
Shoulder Disorders [57]	1	83	DASH-D/S	1	83
Neuromuscular Disease [62]	1	78	ABILHAND	1	78

The diseases were related to spine (41 articles), cranial (six articles), and others (three articles). The most common condition was back pain and cervical spine disorder (21 articles) for which the tools utilized most were SF-12 and SF-36 (nine articles), RMDQ (eight articles), and ODI (five articles). The second most common disease was idiopathic scoliosis (14 articles) for which the most popular tool was SRS-22 (14 articles). The correlation findings between article citation numbers and various parameters are summarized in Table 6.

**Table 6:** Correlation findings between the citation numbers and characteristics for the 50 most cited studies in the neurospine surgical literature that utilized PROMs *Denotes significance, which was determined when p≤0.05

Features	R-value	P-Value	
			Journals' impact factor	0.0518	0.7209	
Articles age (years)	0.4381	0.0015*	
PROMs tool types	0.063	0.6071	
Number of PROM tools	0.0207	0.8865	
PROMs tools translated versions	-0.1427	0.3229	
Clinical diagnoses for using PROMs tools	0.0189	0.8963	
Number of participating patients	0.3603	0.0101*	
Response rate	-0.3439	0.0145*	
Number of authors	-0.0442	0.7605	
Number of centres	-0.2616	0.0665	
Number of countries	0.0617	0.6704	
First author’s country	0.2047	0.1539	

A significant correlation was observed between citation numbers and article age and number of participants (p=0.0015 and p=0.0101, respectively). A positive inverse correlation between citation numbers and response rate (p=0.0145) was also noted. This could be the result of bias related to participant numbers and article age. Studies that reported an equal or lower response rate than the mean (≤82%) compared to a response rate higher than the mean (>82%) had a higher mean number of participants number (570 vs. 250) and were older (19 vs. 17) years. None of the other parameters, including the utilized PROM types, their numbers, or the clinical diagnoses, had any significant association with citation numbers.

Discussion

Only two of the 33 PROM tools identified in this review were generic, which is not unusual. Generic PROMs assess health, disability, and QOL across a wide spectrum of conditions. However, they are less responsive to changes in a specific condition than disease-specific instruments [63]. Churruca et al. [1] found 315 PROMs in the academic and grey literature, of which only 39 were generic. Furthermore, Bohm et al. [3] identified less generic PROMs than joint-specific arthroplasty registries (22 vs. 58). The most utilized and cited tool in this review was SRS-22 (15 articles and 2,869 citations). The tool was developed by Asher et al. in 2003 as a means of assessing QOL in scoliosis [13]. Their paper was the most cited of the selected articles in this review receiving 675 citations. Further, Asher first authored five of the publications in this review [13,20,21,22,41]. SRS-22 is multidimensional and covers five domains, namely, function, pain, mental health, self-image, and management satisfaction/dissatisfaction [64]. The tool was also found to be useful in evaluating QOL after brace treatment [65]. Six of the articles in this review utilized the translated versions of SRS-22 to German [59], Japanese [46], Spanish [36,40], Chinese [35], and Turkish [24]. Monticone et al. [64] evaluated the methodological quality of 17 translated versions of SRS-22 and concluded that some translated versions were advisable (Chinese, Dutch, Italia, Norwegian, and Spanish), while others (Greek, Japanese, Korean, Persian, Thai, and Turkish) should be used with caution.

The second most utilized and cited PROMs were the SF-12 and SF-36 (11 articles and 2558 citations). SF-36 was developed and validated by Ware in 1992 as a generic short-form tool for measuring QOL [66]. It consists of eight QOL domains: physical functioning, role physical, bodily pain, general health, vitality, social functioning, role emotional, and mental health [67]. A substantially shorter version, the SF-12, that was quicker to complete was later introduced. The two versions correlated well with about 90% of the variation in the physical and mental components explained by both [67,68]. SF-12 and SF-36 have been translated into 29 languages [66]. Only one of the articles in this review used the translated version of SF-36 to Italian [51].

The third most utilized and cited PROMs was the RMDQ (nine articles and 1,456 citations). RMDQ was created by Roland and Morris in 1983 and provides a tool for measuring the level of disability experienced by a patient suffering from low back pain [69]. The tool comprises 24 items relating to the degree of disability experienced during activities such as sleeping, standing, walking, sitting, and working [69]. RMDQ has been translated into more than 50 versions [70]. Six of the selected articles in this review utilized the translated versions of RMDQ to German [23,59], Italian [31,51], Japanese [16], and Portuguese [33].

The fourth most utilized and cited PROMs in this review was the ODI (five articles and 852 citations). ODI was developed by Fairbank in 1980 and consists of 10 items that assess the level of pain and interference with several physical activities: sleeping, self-care, sex life, social life, and travelling [71]. Twenty-seven versions of ODI in 24 different languages have been reported [71]. Three of the selected articles in this review utilised the translated versions of ODI to Italian [31], Japanese [16], and Colombian [61]. None of the most cited publications in the neurospine surgical literature utilized the Patient-Reported Outcomes Measurement Information System (PROMIS). The latter was developed in 2004 to validate PROMs for clinical research and practice. PROMIS questionnaires offer a set of short-form versions with a fixed set of questions tailored to individuals and the severity of their symptoms [72]. The PROMIS physical function, pain intensity, pain interference, and pain behaviour were found to have a moderate to strong correlation with ODI, SRS-22, and SF-12 [72].

In this study, 21 articles that received 3,499 citations used PROMs to assess low back pain and cervical spine disease. The most frequently utilized tools were SF-12 and SF-36, RMDQ, ODI, FABQ, and VAS, which conforms with the literature. VAS, ODI, SF-36, and SRS-22 were reported to be amongst the top six tools used in research that was published in five orthopaedic journals over a 10-year period [5]. The list also included the neck disability index (NDI) and the modified Japanese Orthopaedic Association (JOA) [5]. ODI and RMDQ were listed amongst the 13 tools that were used for the assessment of chronic low back pain [6]. ODI, RMDQ, and SRS-22 were mentioned amongst the five most utilized PROMs in spine surgery [7]. The other two are NDI and JOA [7]. The correlation between some of the PROMs used in spine surgery was also examined. A moderate correlation between ODI or RMDQ as a condition-specific outcome and the SF-36 indicating overall health status was observed [73]. ODI was found to be a more adequate measure to evaluate axial pain, rather than referred pain or radiating pain. RMDQ was adequate to measure the health status and evaluate the three types of pain [74].

Fourteen of the selected articles that received 2,781 used PROMs to evaluate idiopathic scoliosis. The most frequently utilized PROMs were SRS-22, SF-36, and RMDQ. In a scoping review, Parent et al. [75] reported that the most used PROMs in five groups of non-operative scoliosis and deformity patients were ODI (37.3%), SRS-22 (34.8%), and SF-36 (20.1%). The studies that used SRS-22 in this review focussed on validating the tool’s discriminating capacity, responsiveness to changes, suitability for the young, minimally important differences after surgery, and the validation of translated versions (Table 2). The findings that all the scoliosis articles in this review used SRS-22 are a reflection of the dominance of SRS-22 as the PROM tool for scoliosis research.

Five of the reviewed articles received 1,589 citations that used PROMs to appraise spinal cord injury (SCI). Only one study used SF-36 [45]. The latter was found to be the most useful generic measure in evaluating the impact of spasticity on QOL in patients with SCI [76]. Only seven of the 50 most cited articles that received 1,369 citations used PROMs for a variety of neurosurgical and neurological diseases. The under-representation of the two fields amongst the studies that employed PROMs is not surprising. The utilization of PROMs in paediatric neurosurgical research has not increased in the last 10 years [9]. It has also been suggested that PROMs that currently feature in the neurosurgical literature may not address the specific outcomes relevant to neurosurgical practice [8]. 

The citation numbers for PROM-utilising high-impact publications in the neurospine surgical literature were found to be impacted by article age, the number of participants, and the response rate. The association between citation numbers and the age of the article and the study population is well recognized [10,77]. The inverse correlation between citation numbers and the response rate was surprising and could have been influenced by participant numbers and article age. The negative association between citation numbers and journals IF is also unusual [10,77]. The lack of correlation between citation numbers and the PROMs types or the clinical diagnoses could relate to the multiplicity of utilized tools and their spread across a wide range of articles that had different diagnoses and citation numbers.

Limitations

There are several limitations to this study. The study was reliant on the precision of the online search engines PubMed and Google Scholar. The search was limited to English literature. It is possible that the translated versions of PROMs were published in other languages. The selection of the 50 most cited studies was based on their citation at a certain point which was likely to change relatively quickly. This could have influenced the selection of utilized PROMs in the lower-impact publications. Relevant studies that utilized PROMs and were published outside the neurospine surgical journals were not included. The review did not necessarily provide a specific solution to which PROMs to be used. The methodological quality of the articles was not assessed.

## Conclusions

A wide range of PROM tools was utilized in the 50 most cited publications in the neurospine literature. The majority were disease-specific rather than generic and targeted particular spine pathologies, of which back pain, cervical spine disorder, and scoliosis were the most frequent. Neurosurgical PROMs were under-represented. Awareness of the PROMs used in high-impact studies may be useful in tool selection in future research. PROMs are useful in standardizing subjective outcomes. Their use in research and clinical settings in any validated language is highly encouraged.
